# Effects of Bonding Agents on Metal-Ceramic Bond Strength of Co-Cr Alloys Fabricated by Selective Laser Melting

**DOI:** 10.3390/ma13194322

**Published:** 2020-09-28

**Authors:** Soo-Yoen Yoo, Seong-Kyun Kim, Seong-Joo Heo, Jai-Young Koak, Joung-Gyu Kim

**Affiliations:** 1 Department of Prosthodontics and Dental Research Institute, Seoul National University Dental Hospital, School of Dentistry, Seoul National University, 101 Daehak-ro, Jongno-gu, Seoul 03080, Korea; sy0502@snu.ac.kr (S.-Y.Y.); heosj@snu.ac.kr (S.-J.H.); young21c@snu.ac.kr (J.-Y.K.); 2 Sense Dental Laboratory, 1104, Seoul Soop IT-Valley, 77, Seongsuil-ro, Seongdong-gu, Seoul 04790, Korea; senselab@naver.com

**Keywords:** cobalt-chromium (Co-Cr) alloy, selective laser melting (SLM), bond strength, metal bonding agent, three-point bending test, fracture failure mode

## Abstract

Bonding agents have been developed to improve bond strength between ceramic and Co-Cr metal. The aim of this study was to investigate the influence of two bonding agents on bond strength of Co-Cr metal fabricated by selective laser melting (SLM). Bond strength was determined by a three-point bending test, and the interfaces of the metal and ceramic, before and after the bending test, were observed by optical microscopy and scanning electron microscopy (SEM) to determine the thickness of the oxide layer and amount of ceramic remaining. To analyze the elemental composition of the bonding agents and fractured surfaces, energy dispersive X-ray spectroscopy (EDS) was used. Co-Cr specimens with bonding agent showed significantly higher bond strength than Co-Cr specimens without bonding agents. The fractured surfaces of most specimens showed mixed failure, but failure mode varied according to bonding agent and fabrication type. Specimens from groups treated with bonding agents had significantly higher remaining ceramic fractions on fractured Co-Cr alloys than specimens from groups that did not receive bonding agent. Mass amounts of silicone (Si) and titanium (Ti) on the fractured alloy surfaces were also different among specimens according to method of fabrication and presence of bonding agent. Together, the results suggest that application of bonding agent to 3D printed Co-Cr metal increases bond strength with ceramics.

## 1. Introduction

To reduce the risk of internal micro-cracking during the cooling phase of ceramic fabrication, porcelain-fused-to-metal (PFM) crowns were developed in the late 1950s. For 60 years, PFM has been the main type of prosthesis material used in dentistry, although recent studies have focused on development and improvement of metal-free esthetic restorations such as zirconia. However, base metal alloys are economical alternatives to gold alloys and zirconia. Furthermore, thinner and longer infrastructures can be fabricated using base metal alloys than zirconia and gold alloys because these base metal alloys have greater rigidity related to their modulus of elasticity [1].

Ni-Cr alloy as a PFM framework was replaced by Co-Cr due to allergic and toxic reactions in the oral cavity. De Melo et al. investigated the bond strength of dental ceramic and demonstrated that it did not differ between Ni-Cr alloy and Co-Cr alloy [2]. Heat-resistant and non-magnetic Co-Cr alloys have high strength and favorable resistance to wear, corrosion, and tarnish. Joias et al. found that the bond strength of ceramic to Co-Cr alloy depends on the alloy composition [3]. Some studies have reported that the bond strength of ceramic to Co-Cr alloys fabricated using the lost wax technique ranges from 35 MPa to 95 MPa, which is an appropriate range for clinical applications [4,5,6].

The Co-Cr framework fabricated by casting is labor intensive and might amplify errors in the manufacturing process. Developments in dental technology over the past few decades have resulted in alternative restoration methods to the conventional lost wax technique for Co-Cr alloys. The first method involves computer-aided design—computer-aided manufacture (CAD-CAM) technology, while the other method is based on selective laser melting (SLM), commonly known as laser sintering. Both techniques are promising for further dental applications due to their cost and labor effectiveness. SLM restoration in particular is less expensive and time-consuming than the conventional lost wax technique without any compromise in quality of the final product. SLM also eliminates internal porosity and provides a single-phase microstructure, thereby preventing galvanic coupling within alloy phases [1]. Co-Cr alloys fabricated by the SLM method exhibit better microstructures and mechanical properties than those fabricated by milling or casting [7]. Although SLM appears to be a very promising technique, many other properties of the resulting materials, such as fit accuracy and metal-ceramic bond strength, need to be investigated in greater detail to validate SLM as a reliable alternative method to the conventional lost wax technique.

The success of metal–ceramic prostheses depends primarily on optimal bond strength between the ceramic and metal components. The bonding behavior of ceramic to metal can be described both chemically and micro-mechanically. Oxide-forming constituents in the metal alloy form an appropriate thickness oxide layer on the metal surface. The veneering ceramic bonds to this oxide surface by a combination of mechanical retention, van der Waals interactions, and chemical bonding with the oxide layer, the latter making the most significant contribution to bond strength between ceramic and metal [8]. Mechanical retention is another important determinant of bond strength. For stronger mechanical bonding, proper metal framework design, adequate veneering ceramic support, and adequate ceramic thickness are important [9]. A higher surface roughness increases metal–ceramic bond strength due to improved mechanical interlocking between the metal and ceramic components. Therefore, sand blasting the metal alloy with airborne particles results in better bonding [10].

A compatible coefficient of thermal expansion (CTE) between ceramic and metal is mandatory for successful PFM prostheses [11,12,13] as the CTE mismatches induce bonding failure. If the contraction of the alloy is greater than that of ceramic, during the cooling phase there will be compressive stress within the ceramic and tensile stress in the metal alloy. This compressive force on ceramic might close the primary flaws and crazing as well as prohibit crack propagation. Therefore, a slightly greater coefficient of thermal expansion of metal substrate than the ceramic is essential. The coefficient of thermal expansion may be influenced by inclusion of minor constituents such as aluminum (Al), Si, Ti within the composition of metal and ceramic. A typical range of the coefficient of thermal expansion for the porcelain is from 13.0 to 14.5 × 10^−6^/°C, whereas that of the metal alloy typically has a slightly higher range of 13.5 to 14.9 × 10^−6^/°C.

Many studies on Co-Cr alloys fabricated by SLM have demonstrated that the bond strength of the ceramic to the SLM Co-Cr metal fulfills the ISO 9693-1 criterion for a minimum acceptable metal ceramic bond strength of 25 MPa. However, few studies have examined the bond strength of ceramic to SLM Co-Cr alloy after thermal aging to reproduce the actual oral environment, and clinicians and technicians often report bonding failure of ceramic to SLM Co-Cr alloys in practice [14,15,16]. Therefore, methods are needed to increase the bond strength of ceramic to Co-Cr alloy fabricated by SLM to withstand the stresses created in the dynamic environment of the oral cavity.

McLean et al. reported that chromium oxide in Co-Cr adversely affected the bond strength by lowering the CTE of ceramic. In contrast, Cr might prevent formation of an excessive oxide layer, which weakens bond strength [17,18]. Isil et al. reported that silicone (Si) coating improved the bond strength and Wang et al. showed that Si_3_N_4_ and Cr coating reduced the thickness of the oxide layer, resulting in stronger bonding [19,20]. Coating of chemical elements such as Au, Si, Ti on the metal substrate has also been shown to improve bond strength between ceramic and metal. Ceramic manufacturers have recently introduced several types of metal bonding agents to improve bond strength between ceramic and metal.

Our aim in this study was to evaluate the bond strength of dental ceramic to Co-Cr alloy fabricated by SLM after thermal aging of specimens to reproduce the oral environment. Additionally, we wanted to verify that commercially available bonding agents for SLM Co-Cr alloys did increase the strength of the bond between ceramic and SLM Co-Cr alloys as claimed by the manufacturers

## 2. Materials and Methods 

**Figure 1:**
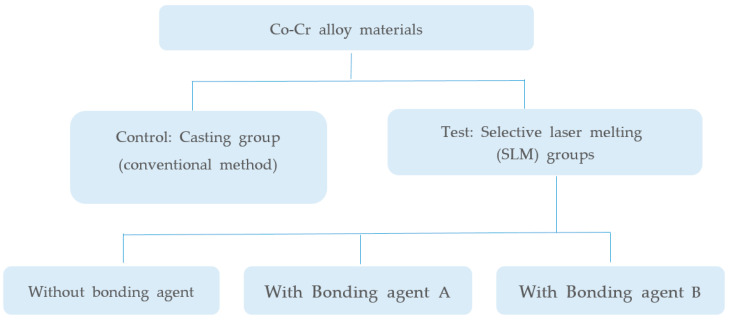
Flow chart of the bond strength experiments.

A flow chart of our experimental design is presented in Figure 1. Co-Cr alloy substrates for metal bond strength tests were fabricated by two manufacturing methods (SLM and conventional casting). SLM-manufactured specimens were divided into three groups based on application of our bonding agent and two commercial bonding agents. Groups and materials used in this study are shown in Table 1.

### 2.1. Specimen Preparation and Analysis of Bonding Agents

#### 2.1.1. Specimen Preparation for Metal-Ceramic Bond Strength Tests

**Figure 2:**
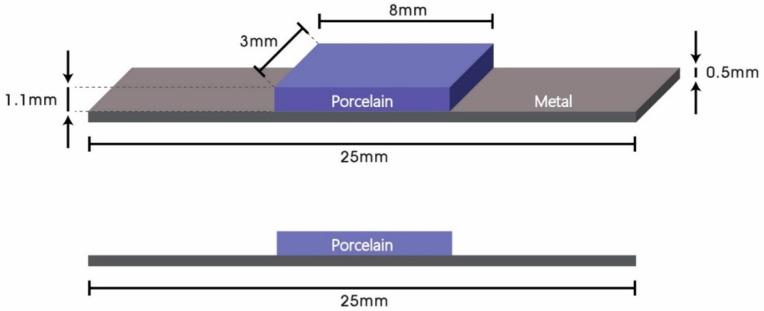
Schematic diagram of metal-ceramic specimens conforming to ISO 9693 guidelines.

In the present study, 20 metal substrates of Co-Cr alloy (Star Loy C, Dentsply Sirona, PA, USA) were fabricated using the lost wax technique, and 60 metal substrates of Co-Cr alloy (Starbond CoS powder, Scheftner Dental Alloys, Mainz, Germany) were fabricated using SLM (Concept Laser Mlab, Concept Laser GmbH, Lichtenfels, Germany). Substrates were fabricated to have a parallelepiped shape with a width of 3 mm, length of 25 mm, and thickness of 0.5 mm according to ISO 9693 specifications. The 60 SLM metal substrates were divided into three groups of 20 specimens each; one without bonding agent (only sandblasted) and one treated with each commercial bonding agent. A single type of dental porcelain with dimensions of 8 × 3 × 1.1 mm was applied to the specimens. An illustration of a specimen used in the present study conforming to ISO 9693 guidelines is shown in Figure 2. Brand names, elemental compositions of the alloys, and manufacturers are provided in Table 1.

Twenty wax models for metal alloy specimens were cast in a high-frequency electric heater (casting machine, Seki Dental, Seoul, Korea). There was no post-heat treatment after cooling at room temperature. SLM specimens were designed using CAD software (3shape CAD, 3shape, Copenhagen, Denmark), and the data were transferred to a three-dimensional printing machine. Production of 60 samples of 30 μm thickness was carried out through melting and binding the Co-Cr-based metal powder (Starbond CoS powder, Scheftner Dental Alloys, Mainz, Germany) in a selective laser sintering device (Concept Laser Mlab, Concept Laser GmbH, Lichtenfels, Germany) layer by layer in accordance with the virtual design. Parameters of the laser system and the firing schedule of the metal frameworks were set-up according to the manufacturer’s recommendations.

#### 2.1.2. Analysis of Bonding Agent Surface

Two metal bonding agents (shown in Table 1) were applied to specimens according to the manufacturers’ instructions. To determine which elements were present in the bonding agents, we analyzed their surfaces using energy dispersive X-ray spectroscopy (EDS).

#### 2.1.3. Application of Veneering Ceramic

To ensure a flat surface without concavity or convexity, all metal specimens were inspected, and the dimensions of samples were carefully measured with calipers (Dial Caliper, Starrett Company, Athol, MA, USA). Before the oxidation process, 80 Co-Cr metal samples were cleaned in an ultrasonic cleaner (BioSonic UC50, Coltène/Whaledent GmbH&Co. KG, Langenau, Germany) with distilled water for 10 min, followed by washing with ethyl alcohol for 10 min to remove surface residue. After oxidation, samples were blasted with 50 μm Al_2_O_3_ particles (Eazimill A11; Vericom, Korea) from a 1 cm distance for 5 s under 4 atm pressure. The center 8 mm of the samples on one surface were measured for ceramic application. Bonding agents A and B were applied to the 40 metal Co-Cr alloy plates produced by SLM before application of opaque porcelain according to the manufacturer’s instructions. Opaque porcelain was applied at the same place and with the same dimensions on 80 samples. Ceramic firing of the samples was performed in a ceramic furnace (Programat p500, Ivoclar Vivadent, Schaan, Liechtenstein) following the manufacturer’s instructions (shown in Table 2). Dimensions were measured with a micrometer (Dial Caliper, Starrett Company, Athol, MA, USA) to ensure that all fired samples had dimensions of 8 × 3 × 1.1 mm.

To determine the thickness of the oxide layer, we examined the metal ceramic bonding surface by field emission scanning electron microscopy (SEM) (ThermoFisher Scientific, Apreo S, MA, USA) laterally after specimen surfaces were sputtered with an Au-layer (Polaron SC 7620 Sputter Coater, Quorum Technologies Ltd., Kent, GB, UK) due to the low conductivity of porcelain.

### 2.2. Bond Strength Test and Characterization of the Co-Cr Metal and Ceramic Interface

#### 2.2.1. Metal–Ceramic Bond Strength Test

**Figure 3:**
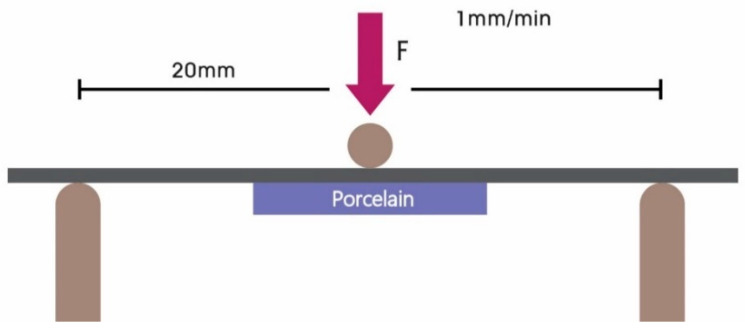
Schematic diagram of the three-point bending test.

The three-point bending test performed according to the ISO 9693 standard is widely used to measure metal–ceramic bond strength (shown in Figure 3). Ceramic metal specimens were tested in a desktop universal testing device (TW-D102, Taewon Tech, Seoul, Korea) with a head displacement speed of 1.0 mm/min. Samples with ceramic superstructures were placed face down on the device, which was adjusted to a 20 mm distance between the two supports. The load was adjusted to proceed at the rate of 1 mm/min vertically to the center of the metal surface on the specimens until breaking of the metal-ceramic interface bond. The force (N) that caused crack initiation or fracture on the metal-ceramic interface was recorded by software (Trapezium X Material Testing Operation Software, Shimadzu, Kyoto, Japan) on a computer connected to the universal testing device.

Metal–ceramic bond strength (σ(MPa)) of all specimens was calculated as σ (N/mm^2^) = *k* × *F*, where *k* is a constant related to thickness and the modulus of elasticity of the Co-Cr alloy, and F is the load (in N) that causes de-bonding between metal and porcelain. In this study, the *E*-moduli of Co-Cr specimens fabricated by conventional casting and SLM were 200 and 197.5 GPa, respectively.

Before the metal–ceramic bond strength test, thermal cycling was performed to simulate the oral environment [21]. All specimens were stored in distilled water at 37 °C. Thermal aging was performed by thermo-cycling using a thermo-cycling machine (Taewon Tech, Seoul, Korea) for 5000 cycles between 5° and 55 °C (dwell time of 30 s). This procedure corresponds to a five-year period of oral temperature conditions [22]. To avoid sample confusion during thermal cycling, samples from each of the four groups were placed in individual mesh bags.

#### 2.2.2. Characterization of the Metal–Ceramic Interface

After the three-point bending test, crack propagation between metal and ceramic was analyzed by SEM (magnification 1000×, 200×). Subsequently, the metal-ceramic surface was manually de-bonded and the fractured surface was examined using SEM (magnification 300×) and then analyzed with energy dispersive X-ray spectroscopy (EDS) to detect the elemental distribution on the fractured surface. An optical microscope (Olympus SZX10, Japan, magnification 8×) and software (Image J, NIH, MD, USA) were used to analyze the failure mode and area fraction of adherent ceramic after de-bonding. Three types of failure mode were considered: adhesive, where less than 20% of the bonding area was covered by remaining ceramic in the Co-Cr substrate surface; mixed, where more than 20% but less than 80% ceramic was present; and cohesive, where more than 80% of the alloy surface was covered by remaining ceramic [23].

### 2.3. Statistical Analysis

Statistical tests were performed using SPSS (IBM SPSS statistics 22, IBM, NY, US) software for Windows. The Levene test was applied to assess the equality of variances, while bond strength data and the Si ratio of fractured surfaces were analyzed in independent groups using one-way analysis (ANOVA). Probability values of *p* < 0.05 were considered to be statistically significant. Multiple comparisons were made by Tukey’s adjustment test. 

## 3. Results

### 3.1. Analysis of Bonding Agent Surface

Table 3 shows the EDS results for the bonding agents used in this study. The two bonding agents had similar element compositions but different mass percentages of the elements. Bonding agent B had much more titanium (Ti) than bonding agent A (Table 3), whereas bonding agent A had more Si than bonding agent B.

### 3.2. Metal–Ceramic Bond Strength and Characterization of the Metal–Ceramic Interface

#### 3.2.1. Metal–Ceramic Bond Strength

The mean bond strengths of the cast and SLM specimens without bonding agent were 32.21 ± 6.88 MPa and 35.29 ± 6.57 MPa, respectively. SLM Co-Cr specimens to which bonding agent was applied had higher bond strength values of 38.38 ± 8.11 MPa (bonding agent A) and 42.56 ± 5.21 MPa (bonding agent B). All samples with bonding agents had significantly higher bond strength than samples without bonding agent. There was no difference in bond strength according to fabrication method. All specimens with bonding agent had a much higher bond strength than the 25 MPa required by ISO 9693. Bond strengths in the various specimen groups are summarized in Table 4.

#### 3.2.2. Metal–Ceramic Interface Examination

**Figure 4:**
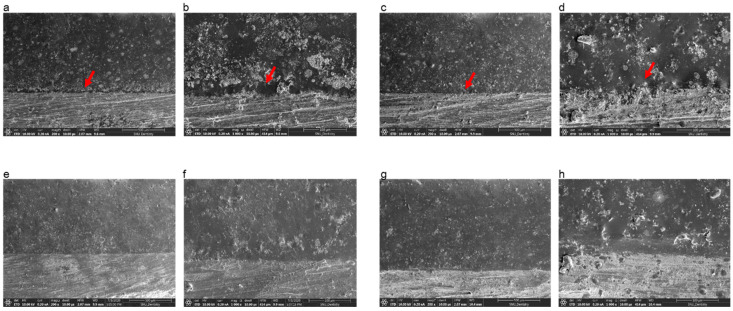
Scanning electron microscopy images of the Co-Cr and ceramic interface after sputtering with Au due to the low conductivity of ceramic. (**a**) Casted specimen, magnification 200×; (**b**) casted specimen, magnification 1000×; (**c**) SLM-fabricated specimen without bonding agents, magnification 200×; (**d**) SLM-fabricated specimen without bonding agents, magnification 1000×; (**e**) SLM-fabricated specimen with bonding agent A, magnification 200×; (**f**) SLM-fabricated specimen with bonding agent A, magnification 1000×; (**g**) SLM-fabricated specimen with bonding agent B, magnification 200×; (**h**) SLM-fabricated specimen with bonding agent B, 1000×. The red arrows indicate the thick oxide layer that looks like a gap between ceramic and metal.

Figure 4 shows cross sections of the metal–ceramic specimens before the three-point bending test. In specimens manufactured by casting or SLM that did not receive bonding agent, a thick oxide layer was present between the ceramic and metal; however, specimens fabricated by SLM that received either bonding agent A or B had intimate connections between the metal and ceramic components.

**Figure 5:**
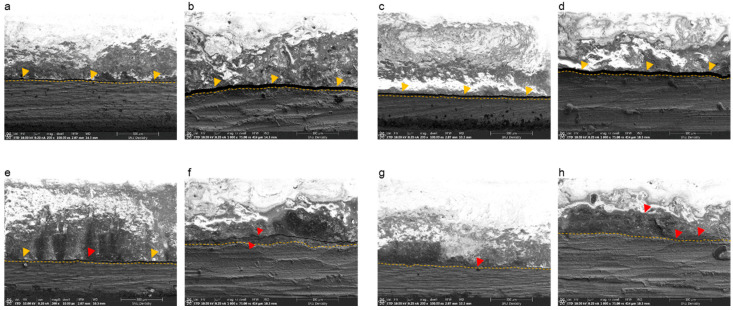
SEM images of specimens after the three-point bending test and before manual detachment of the ceramic portion. (**a**) Casted specimen, magnification 200×; (**b**) casted specimen, magnification 1000×; (**c**) selective laser melting (SLM)-fabricated specimen without bonding agents, magnification 200×; (**d**) SLM-fabricated specimen without bonding agents, magnification 1000×; (**e**) SLM-fabricated specimen with bonding agent A, magnification 200×; (**f**) SLM-fabricated specimen with bonding agent A, magnification 1000×; (**g**) SLM-fabricated specimen with bonding agent B, magnification 200×; (**h**) SLM-fabricated specimen with bonding agent B, magnification 1000×. Dotted lines represent the surface of the Co-Cr alloy. Yellow triangles indicate crack propagation and the large gap between the ceramic and metal layers. Red triangles indicate incomplete crack propagation.

Cross-sectional images of the metal–ceramic specimens after the three-point bending test are presented in Figure 5. For cast and SLM specimens without bonding agents, a large gap was present, and cracks propagated smoothly along the metal-ceramic interface without adherent ceramic (Figure 5a–d). In contrast, for SLM specimens with bonding agent A or B, cracks spread roughly with some remaining opaque ceramic and absence of cracks in some portions of the interface (Figure 5e–h).

#### 3.2.3. Observation of the Fractured Surface

**Figure 6:**
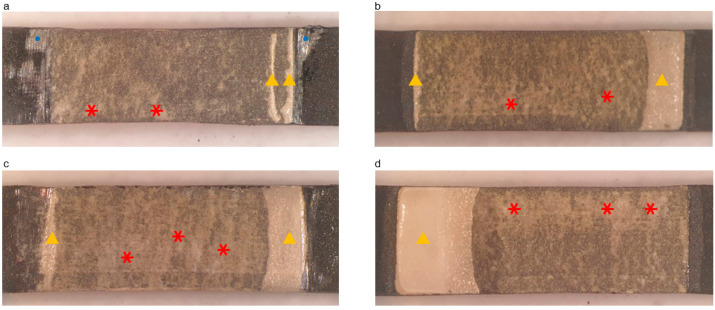
Optical microphotographs of Co-Cr alloy surfaces after de-bonding with ceramic (magnification 8×). (**a**) Casted specimen; (**b**) SLM-fabricated specimen without bonding agent; (**c**) SLM-fabricated specimen with bonding agent A; (**d**) SLM-fabricated specimen with bonding agent B. Yellow triangles represent adherent ceramic, and red asterisks indicate remnant wash opaque. Blue circles indicate remnant sputtered Au.

Figure 6 shows the Co-Cr substrate surfaces of specimens after de-bonding from the ceramic. After the three-point bending test, cracked specimens were manually de-bonded to analyze the remaining ceramic area proportions on the metal surface. Visual inspection and optical microscopy were used to observe the fractured surfaces. There was more adherent ceramic on the alloy surface in the SLM groups with different bonding agents than the other groups.

Failure mode was classified as cohesive (fracture inside ceramic layers), adhesive (fracture interface between ceramic and metal), or mixed (cohesive and adhesive failure) by analyzing the ceramic remnants. The SLM with bonding agent B group showed no adhesive failure and some cohesive failure. Fracture modes are presented in Table 5. The mean amount of ceramic remaining was significantly larger in groups with bonding agents than in those without. Furthermore, there were significant differences in fracture modes between the two commercial bonding agents.

**Figure 7:**
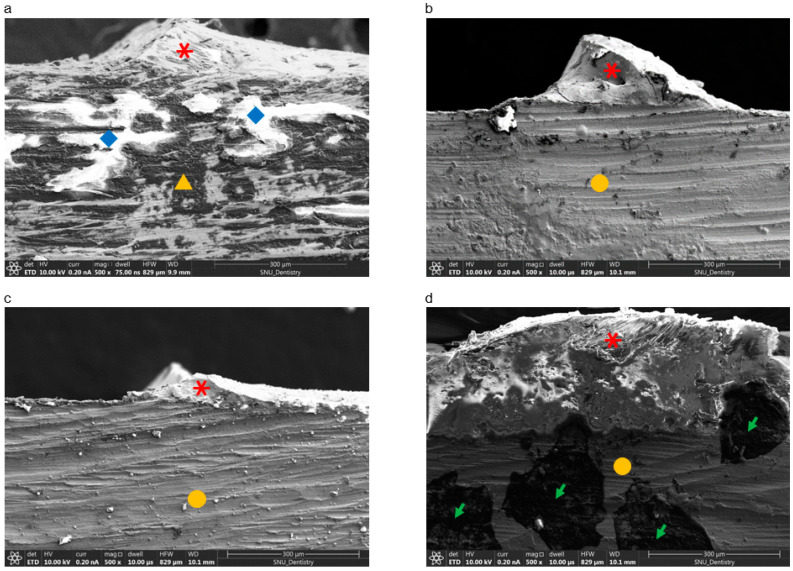
Scanning electron microscopy images (300×) of specimens after de-bonding of ceramic from the Co-Cr alloy surface. (**a**) Casting group; (**b**) SLM-fabricated specimens without bonding agent; (**c**) SLM-fabricated specimens with bonding agent A; (**d**) SLM-fabricated specimens with bonding agent B. Red asterisks represent adherent ceramic. Yellow triangles show Co-Cr substrate fabricated by casting, and yellow circles indicate Co-Cr alloy fabricated by SLM. Blue diamonds indicate wash opaque from ceramic on the Co-Cr substrate, while green arrows indicate remnant Au from sputtering.

SEM images (magnification 300×) of remnant ceramic on the Co-Cr metal are shown in Figure 7. White areas represent adherent opaque and dentine ceramic on the alloy (light due to the low electrical conductivity of the ceramic), while gray areas indicate Co-Cr alloy (dark due to the high electrical conductivity of the alloy). SLM-fabricated specimens with bonding agent B showed more remnant ceramic than the other groups.

**Figure 8:**
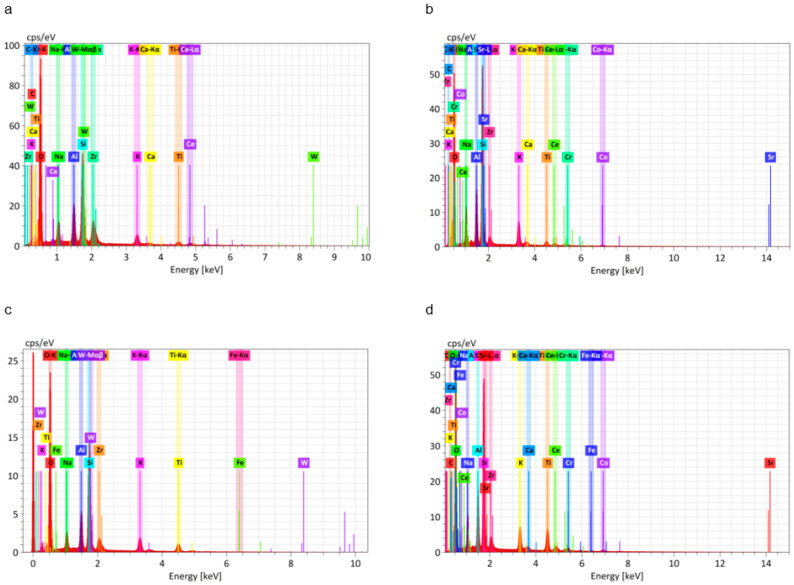
EDS analysis of the fractured Co-Cr and ceramic interface. (**a**) Casted group specimens; (**b**) SLM-fabricated specimens without bonding agent; (**c**) SLM-fabricated specimens with bonding agent A; (**d**) SLM-fabricated specimens with bonding agent B.

EDS analysis results for the fractured surfaces are presented in Figure 8 and Table 6. Based on amount of Si present in representative SEM micrographs, the SLM group with bonding agent B had more adherent ceramic than the other groups.

## 4. Discussion

Bond strength is determined by various factors including mechanical and micromechanical retention, van der Waals interactions and chemical bonding in the oxide layer, and appropriate CTE differences between ceramic and metal components [8,9,10,11]. To evaluate the bond strength between ceramic and metal, we fabricated Co-Cr samples by the conventional lost wax casting method, which has been verified to be suitable for fabrication of specimens for use in a clinical setting, and the SLM method, a newer fabrication method. All metal specimens in our study were sandblasted with 50 um Al_2_O_3_ particles for 5 s to control surface roughness. Previous studies reported that airborne-particle abrasion with 50 µm alumina particles improved the shear bond strength of porcelain to Co-Cr alloy [24,25,26] by increasing the surface roughness of the metal, facilitating micromechanical retention of the porcelain and enhancing the wettability of porcelain on metal [25,27]. Opaque porcelain flows into the microgrooves created by sandblasting on the surface of the alloy and mechanically interlocks into the undercuts [11]. All specimens in this study were abraded with airborne particles, avoiding differences in mechanical retention among SLM fabrication groups (the exception to this is the casted group, which had different metal properties). In our study, the main determinants of bond strength were compressive bonding due to differences in CTE between metal and ceramic and chemical bonding within the oxide layer.

Differences in CTE values induce stress and principally affect the interfacial bond strength that causes ceramic de-lamination or cracking, emphasizing the importance of a compatible CTE between veneering porcelain and Co-Cr alloy. It is essential that the metal substrate has a slightly greater CTE than the ceramic. The CTE can be influenced by minor constituents present in the metal and ceramic. The bonding agents we used in this study were developed to compensate for differences in CTE value between ceramic and metal. Our finding that the specimens to which these bonding agents were applied had higher bond strengths than specimens that did not receive application of these bonding agents indicates that the bonding agents increased bond strength.

Deviations in in vitro and in vivo performances of restorations can result in differences in reported metal–ceramic bond strengths [28]. It is impossible to simulate intraoral conditions perfectly due to all the factors necessary for fatigue-related failures in dental prostheses. In this in vitro study, we attempted to simulate the complexity of the oral environment in terms of temperature and moisture. Therefore, all specimens in this study were subjected to thermal cycling, which can potentially weaken metal–ceramic bonding [24,29,30]. The thermal aging procedure we used was based on previous studies; in particular, the lowest temperature for thermal cycle tests should be 5 °C and the highest 55 °C, with an average retention time of 30 s [24,31,32,33]. All samples in this study underwent 5000 cycles of exposure to 5 °C and 55 °C in accordance with ISO/TS 11,405 recommendations. Thermal cycling tests induce repeated stresses at the interface of two materials. Such stresses arise due to differences in thermal expansion coefficients of the two materials and can lead to adhesion losses [24].

We examined bond strength by three-point bending testing using a universal testing machine. Despite lack of an ideal testing method [34], we used the three-point bending test in this study to determine metal-to-porcelain bond strength because metal and ceramic bond strength tests should be quantifiable, reproducible, and easy to perform [35]; in addition, this test is recommended by the American Dental Association Council on Dental Materials, Instruments, and Equipment.

We found that ceramic and metal bond strengths differed slightly according to fabrication method but without statistical significance. In a similar study, Han et al. reported that bond strength values of samples produced with 3D printer were higher than those produced by the traditional casting method or CAD/CAM [23]. A previous study also reported that SLM metal alloy showed better mechanical microstructure and properties than cast alloy [36], consistent with our findings that the bond strength of specimens fabricated using SLM alloy were slightly higher than those made by casting Co-Cr alloy.

Notably, all Co-Cr substrates with bonding agent showed significantly higher bond strength than substrates without bonding agent. This supports the suggestion of the manufacturers that metal bonding agents affect bond strength by reducing the thickness of the oxide layer and compensating for differences in coefficient of temperature expansion between ceramic and Co-Cr alloy. A previous study reported that use of a metal bonding agent increased the Weibull modulus [37]. This means that the presence of bonding agent narrowed the scatter of values of bond strength; in other words, a more reliable interface between ceramic and metal was produced. A related bond strength study reported a significantly higher bond strength between ceramic and Co-Cr alloy when a metal bonding agent was used [38]. The present study differs from previous studies in that we subjected specimens to thermo-cycling to simulate the oral environment; nevertheless, all specimens with bonding agents had values that were remarkably higher than the minimum 25 MPa value required by ISO 9693 specifications. Some specimens without bonding agents showed inappropriate bond strength regardless of fabrication method.

We investigated the fracture mode between ceramic and metal after the three-point bending test. Previous studies have reported that mixed type failure mode is the most frequent [25,34]. Conversely, another study found a mixed mode of failure in specimens fabricated with the casting technique, while specimens fabricated by the SLM technique had either mixed or adhesive mode of failure [15]. Han et al. reported all adhesive failures in their study regardless of fabrication technique [23].

Under an optical microscope, most specimens with bonding agent B had an opaque layer and ceramic on the surface of the metal, suggesting mixed and cohesive failure mode, which was not seen in other groups. Specimens without bonding agents showed mostly adhesive failure mode, implying that cracks occurred between the metal and ceramic, and that there may have been some mixed failure modes [39]. Adhesive failure is usually not an ideal situation because it indicates a lower bond strength between metal and ceramic than that within the ceramic interior; this requires less destructive force to separate metal and ceramic [40]. In addition to the bond strength results reported here, fracture surface analysis results indicated that use of bonding agents increased the bond strength between ceramic and metal.

EDS analysis showed that elements of porcelain, such as aluminum (Al), Si, and Ti, were present on the fractured Co-Cr alloy surfaces. The presence of Al might be due to remnants of the Al_2_O_3_ air-particle abrasion process and/or porcelain (opaque layer) adhered to the alloy substrate. In our study, as Al compositions among groups were similar, we concluded that most of the Al was contributed by remnant Al_2_O_3_ particles after air-particle abrasion.

Si-containing metal bonding agents absorb excessive oxides that form on the alloy surface during porcelain firing [29,41]. As shown in Table 3, the bonding agents used in our study contained Si to improve bond strength by regulating the thickness of the oxide layer. The Si present on EDS after the stress test indicated remnant ceramic. The amount of Si on the fractured surfaces was greatest for SLM-fabricated alloy with bonding agent B (Figure 7 and Table 6), indicating that specimens in this group had the strongest bond strength in our study.

We used Ti-containing bonding agents in this study (Table 3) as they act as oxygen scavengers to protect the alloy surface from progressive accumulation of an excessive oxidation layer with repetitive firing cycles [27,41,42]. A previous study suggested that titanium–ceramic adhesion involves a chemical reaction between Ti and Si, resulting in formation of a new Ti_5_Si_3_O phase [43]. Chemical bonding with Ti is possible because of diffusion of porcelain components into titanium oxides during firing [44,45]. This phenomenon could be partially responsible for the increased metal–ceramic bond strength that we observed with bonding agent on the Co-Cr metal substrate. Different commercial bonding agents affect bond strength between ceramic and metal components differently; however, a bonding agent that contains Ti can moderate strong oxidation of the metal surface and increase bond strength. In other words, the bonding agent can function as a chemical bridge between ceramic and metal.

Lu et al. linked the dynamic functional theory (DFT)-predicted adhesive energy at solid/liquid interface and cohesive energy at liquid/liquid interface with DFT atomic force microscope (AFM)-predicted force of adhesion through the Young–Dupré equation and established the basis of quantum surface wettability theory by combining two independent atomic-level quantum physics simulation methodologies [46]. They used quantum physics via density functional theory simulation [47] to directly evaluate how the surface wettability of graphite is influenced. The wettability of ceramic to metal examined higher, where an active element, e.g., Al, Zr and Ti, alters the surface chemistry of the ceramic by the formation of intermediate reaction layer and lowers the wetting angle on the ceramic [48]. In our study, SLM-fabricated with bonding agent group B showed highest values of Al and Ti (shown in Table 6). This physical property may also have effects on the positive increase of the bond strength between ceramic and Co-Cr alloy. Quantum chemical calculations at DFT level will help improve interpretation of the results in further research.

Prior to performing three-point bending tests, we inspected the ceramic and metal bonding surfaces by SEM. As shown in Figure 3, for specimens produced by casting and SLM without bonding agent, there was a thick oxide layer which looked like a gap between the metal and ceramic in which ion-covalent bonds dominated. According to the manufacturers, bonding agents aim to close (or adequately reduce) this gap. This results in a corresponding increase in bond strength, consistent with our findings (Table 4). Even though differences in the main components of commercial bonding agents can result in differences in bond strength, we observed no significant differences between the two commercial bonding agents in this study. Therefore, we recommend using either of these two bonding agents to increase bond strength. 

In this study, Concept Laser was used to print the Co-Cr alloy by SLM method with Star bond CoS powder and configured to build layers of 30 µm thickness. The study conducted by de Melo et al. found that rather than differences in layer thicknesses, variations in operating principles of devices used in production of metal and differences in the contents of alloy powders significantly affect metal-to-porcelain bonding [49]. Therefore, different results would be observed if a different powder and/or printing device were used. Additionally, our study did not involve a fatigue procedure, therefore results remain optimistic to a certain extent, as physiochemical changes in dental materials attribute to a dynamic oral environment. Further study reproducing oral conditions based on thermal and mechanical cycling process should be considered. 

Regardless of the limitations of this study, we can apply bonding agents to increase the bond strength between ceramic and Co-Cr SLM alloy in the era of upcoming 3D printing. As we verified, the bond strength values for the groups with application of bonding agents were significantly higher than groups without it thanks to the oxide layer formation of appropriate thickness and compensation for differences in CTE value. Therefore, by using bonding agents, we assumed the long span prostheses which might contain more internal stress and the implant prostheses which do not have any capability to absorb excessive loading due to the lack of periodontal ligament can have more advantages. For greater bond strength between ceramic and Co-Cr alloy, the frequency of the chipping or delamination of ceramic would be remarkably reduced.

## 5. Conclusions

We found that use of both commercial bonding agents significantly increased the bond strength between ceramic and SLM-produced metal alloys. All specimens from the groups in which bonding agents were used had a bond strength higher than 25 MPa, which is clinically acceptable. Although the fractured surfaces of specimens after three-point bending tests showed different characteristics depending on type of bonding agent used (there was no unfavorable adhesive failure for bonding agent group B; whereas, 20% of bonding agent group A showed adhesive failure), both commercial bonding agents we evaluated improved bond strength, indicating suitability of use of either bonding agent. This study does have its limitations, but we are able to determine that the use of bonding agent between ceramic and SLM would be helpful for clinicians to make long-term sustainable long-span prostheses of supporting strut that are fabricated by SLM.

## Figures and Tables

**Table 1:** Specifications of the alloys, ceramic, and bonding agents used in this study.

Material	Material Type	Brand Name	Composition (wt%)	CTE (× 10^−6^ K^−1^)	Manufacturer
**Co-Cr alloy**	**Metal ingots: casting**	Star Loy C	Co 59.4%, Cr 24.5%, W 10%, Nb2%, V 2%, Other (Mo, Si, Fe) ≤ 1%	14.6~14.9	Dentsplysirona, PA, USA Scheftner dental alloys
	**Metal powder: SLM**	Starbond CoS powder	Co 59%, Cr 25%, W 9.5%, Mo 3.5%, Si 1%, Other (C,Mn,Fe,N) ≤ 1%	14.4	
**Ceramic**	**Dental ceramic**	Vintage MP powder	SiO_2_ 55–60%, Al_2_O_3_ 10–16%,	13.6–15.2	Shofu, Dental GmbH
			K_2_O 5–11%, Na_2_O 2–16%		Japan
**Bonding agent**	**Metal bonding agent**	Creation Willi Geller	proprietary	13.3	Hersteller,
					Meiningen, Austria
		Matchmaker CTE buffer	proprietary	unmeasurable (≤14.4)	Davis Schottlander
					Letchworth, Herts, UK

CTE: Coefficient of thermal expansion.

**Table 2:** Firing schedules for the veneering ceramic.

	Pre-Heating Temperature (°C)	Drying Time (min)	Heating Rate (°C/min)	Final Temperature (°C)	Holding Time (min)
Liner (primer)	500	5	55	980	1
First opaque	500	5	55	960	1
Second opaque	500	5	55	940	1
Dentin	500	5	55	920	1
Glaze (self-glaze)	500	5	55	900	1

**Table 3:** Compositions and masses of the bonding agents used in this study.

Mass Norm (%)	C	O	Na	Al	Si	K	Ti	Cr	Mn	Fe	W
Bonding agent A	2.21	55.54	1.03	5.25	20.56	3.2	7.21	0	0	1.83	3.17
Bonding agent B	1.44	46.53	0.98	4.33	16.08	2.85	21.16	0.84	1.01	2.37	2.41

**Table 4:** Metal–ceramic bond strength (mean ± standard deviation) and passing rate (≥25 MPa).

Groups	Bond Strength (Mpa)	Passing Rate ≥ 25 Mpa (%)	*p*-Value
Casting	32.21 ± 6.88 a	75	vs. SLM-fabricated with bonding agent A: 0.002
			vs. SLM-fabricated with bonding agent B < 0.001
SLM-fabricated	35.29 ± 6.57 a	75	vs. SLM-fabricated with bonding agent A: 0.027
without bonding agent			vs. SLM-fabricated with bonding agent B: 0.001
SLM-fabricated	38.38 ± 8.11 b	100	vs. Casting group: 0.002
with bonding agent A			vs. SLM-fabricated without bonding agent: 0.027
SLM-fabricated	42.56 ± 5.21 b	100	vs. Casting group < 0.001
with bonding agent B			vs. SLM-fabricated without bonding agent: 0.001

Different lowercase letters (a,b in the table) indicate significant differences (*p* < 0.05).

**Table 5:** Results of failure mode analysis.

Groups	Mixed Failure	Cohesive Failure	Adhesive Failure	Mean Ceramic Fraction (%)
Casting	2	1	17	19.6 ± 17.12 a
SLM-fabricated without bonding agent	8	0	12	20.4 ± 10.22 a
SLM-fabricated with bonding agent A	16	0	4	40.5 ± 18.77 b
SLM-fabricated with bonding agent B	14	6	0	59.65 ± 21.24 c

Different lowercase letters (a,b,c in table) indicate significantly different groups (*p* < 0.05).

**Table 6:** EDS analysis results for the fracture surfaces (mean ± standard deviation).

Specimen Groups	O	Al	Si	Zr	Ti
Casting	43.18 ± 1.00	7.53 ± 1.00	18.91 ± 0.84	5.23 ± 2.19	2.34 ± 4.84
SLM-fabricated without bonding agent	37.69 ± 7.58	7.65 ± 1.13	16.37 ± 1.94	2.2 ± 3.01	3.04 ± 1.20
SLM-fabricated with bonding agent A	53.32 ± 5.73	7.02 ± 1.61	17.21 ± 2.73	3.29 ± 0.56	2.53 ± 4.39
SLM-fabricated with bonding agent B	49.18 ± 0.17	7.99 ± 2.33	22.56 ± 2.54	3.21 ± 1.44	5.8 ± 1.73
